# Advances in muscle imaging for Emery-Dreifuss muscular dystrophy

**DOI:** 10.1186/1750-1172-10-S2-O26

**Published:** 2015-11-11

**Authors:** Nicola Carboni

**Affiliations:** 1 Division of Neurology, San Francesco Hospital of Nuoro, Nuoro, Italy

## 

Laminopathies are a heterogeneous group of disorders related to alterations on genes coding for proteins of the nuclear envelope. Among these clinical entities, there are several diseases affecting mainly the cardiac and skeletal muscles. These disorders include forms with a selective cardiac compromise and muscular dystrophies (autosomal and X-linked forms of Emery-Dreifuss muscular dystrophy, Limb girdle muscular dystrophy 1B, *LMNA*-related congenital muscular dystrophy and other rare clinical entities). We performed imaging studies on a large cohort of subjects bearing either *LMNA* or *EMD* gene mutations; each patient enrolled displayed variable compromise on posterior legs’ muscles, ranging from mild compromise on soleus and medial head of gastrocnemius to overt alterations on soleus, medial head of gastrocnemius[1,3]. Of note, we saw that even subjects presenting with clinically selective cardiac compromise displayed, on imaging studies, variable alterations on skeletal muscles. This findings showed a continuum in skeletal muscles compromise among different phenotypes related to *LMNA* or *EMD* gene mutations and lead to hypothesize a common mechanism in the process of damage of skeletal muscles fibers.
